# Impact of NIHR HTA Programme funded research on NICE clinical guidelines: a retrospective cohort

**DOI:** 10.1186/s12961-015-0025-8

**Published:** 2015-08-22

**Authors:** Sheila Turner, Sheetal Bhurke, Andrew Cook

**Affiliations:** National Institute for Health Research, Evaluation, Trials and Studies Coordinating Centre (NETSCC), University of Southampton, Alpha House, Enterprise Road, Southampton, SO16 7NS UK; University of Southampton, Wessex Institute and University Hospital Southampton NHS Foundation Trust, University of Southampton, Alpha House, Enterprise Road, Southampton, SO16 7NS UK

**Keywords:** Impact, Health Technology Assessment, NICE, Clinical guidelines

## Abstract

**Background:**

It is vitally important that there is a connection between health research and clinical practice. Indications as to the impact of the research on evidence-based practice and policy can be obtained by tracking the use of outputs of health research, especially its use in clinical guidelines (CGs). This study aims to assess the proportion of National Institute for Health and Care Excellence (NICE) CGs citing National Institute for Health Research Health Technology Assessment (NIHR HTA) studies and the impact of evidence from those studies on the included NICE CGs.

**Methods:**

This is a retrospective cohort study assessing the proportion of NICE CGs from all NICE CGs issued between April 2001 and April 2012, which cited evidence from studies funded by the NIHR HTA Programme and the impact of those studies on the CGs as the primary and secondary outcome measures.

**Results:**

Of the cohort of NICE CGs (n = 122), 3 (2%) CGs were based on previous NIHR HTA reports and would not have been issued in that form without those NIHR HTA studies, 90 (74%) included evidence from NIHR HTA studies, and 29 (24%) did not include evidence from NIHR HTA studies. The impact of NIHR HTA evidence on NICE CGs varied in the type and quantity of data used.

**Conclusions:**

Findings suggest that NIHR HTA funded research impacts on clinical guidance from NICE and hence is well connected to both clinical practice and policy.

## Background

It is vitally important that there is a connection between health research and clinical practice [1‐3]. It is also of great importance to research funders to assess the impact of the research they fund on clinical practice and policy. The National Institute for Health and Care Excellence [4] (NICE) publishes several types of publication [4], including clinical guidelines (CGs) which recommend appropriate treatment and care for people with specific conditions or diseases. The organisation has developed an international reputation, its guidance frequently being consulted (as judged by high numbers of visits to the website) or adopted by those in other countries [5]. The recommendations in NICE CGs are prepared by groups of experts (clinicians, lay members, and patients) who examine the available evidence on particular diseases or clinical areas. The evidence used may include systematic reviews, technology assessment reports, and findings from clinical trials, and is moderated by the clinical and patient experience of members of the guideline groups. Use of this evidence exemplifies the concept of evidence-based practice and aims to encourage practitioners to employ similar high standards of care [6].

The National Institute for Health Research Health Technology Assessment (NIHR HTA) Programme [7] produces independent research information about the effectiveness, costs, and broader impact of healthcare treatments and tests, for those who plan, provide, or receive care in the National Health Service (NHS). As part of the NIHR journals library [8], each NIHR HTA funded project publishes an open access report in the NIHR journal *Health Technology Assessment* [9]. This journal is peer reviewed, indexed on Medline, and is freely available online via the NIHR journals library website [8]. Additionally, researchers funded by NIHR HTA are also strongly encouraged to disseminate their findings through other peer reviewed journals [10] and non-academic routes aimed at both professional communities and the general public. Research funded by the NIHR HTA programme includes primary research, systematic reviews, evidence syntheses, and technology assessment reports, and acts as a bridge between evidence and policymaking, providing NICE with accessible and evidence-based information to guide their decisions. Conversely, the NIHR HTA programme uses knowledge gaps identified by NICE to inform areas of future research. It should be noted, however, that the two organisations are separate and each operates independently of the other.

It is extremely useful and important to track the output of health research [11,12], especially its use in clinical guidelines [13‐15]. There has been much debate about how the impact of research may be defined or measured [16,17]. Citation in clinical guidelines can provide a broad indication that the research has been included and therefore has made a contribution, and so may be considered as having had impact on policy and evidence-based practice [14]. It is widely recognised that an important element of impact of research is influence on policymakers and clinical guidelines [15], and it is important for research funders to know the degree to which their funded research is used in order to help shape future funding strategies.

A study undertaken by Alderson and Tan in 2011 [18] investigated the number of Cochrane Reviews cited in NICE CGs and found that 81% of NICE CGs cited these, but that not all Cochrane Review Groups were represented in the reviews cited. Similarly, this study seeks to investigate the number of NICE CGs citing NIHR HTA funded studies and to assess the impact of those studies on NICE CGs.

## Methods

### Phase 1

We identified all NICE CGs issued between April 2001 and April 2012 through an online search of the NICE website [4]. We excluded all CGs which had been withdrawn or superseded by subsequent CGs. The reference section for each included NICE CG (including appendices if they contained reference sections) was independently searched by two reviewers (ST and SB) to identify NIHR HTA research cited. Search terms used to identify NIHR HTA research included ‘HTA’, ‘Health Technol Assess’, ‘Health Technology Assessment’, ‘NCCHTA’, and ‘technology appraisal’. We defined NIHR HTA research outputs as publications in the NIHR HTA journal or other peer reviewed publications arising from projects funded by the NIHR HTA. Investigators funded by NIHR HTA are required to inform the funders of all output from their projects, including articles in peer reviewed journals (other than publications in the NIHR journals library). Notifications of these publications are recorded in an internal database. We identified peer reviewed journal publications arising from NIHR HTA funded studies by searching this internal database and matched these titles to citations in the CGs. However, not all investigators inform the NIHR HTA when they publish peer reviewed articles, so we also searched the reference sections of the CGs using author names and key words from relevant NIHR journals, and checked articles identified for information as to which organisation had funded the study, recording those funded by NIHR HTA. We then calculated the percentage of NICE CGs citing NIHR HTA funded studies.

### Phase 2

In the second phase of this study we classified the cohort of NICE CGs according to the extent to which they were influenced by NIHR HTA studies using the criteria described in Table 1. Category 1 included CGs which were based on a previous NIHR HTA journal publication(s) and could not have been issued in the current form in absence of evidence from the NIHR HTA study(s). Category 2 included CGs which either reviewed NIHR HTA study(s) in detail, reproduced evidence table(s) from NIHR HTA study(s), or had one or more sections drawing evidence from NIHR HTA study(s). Examples which clarify the categorisations are given in the results section. Category 3 included all the CGs where no NIHR HTA study(s) were cited.

**Table 1:** **Impact of NIHR HTA evidence on NICE Clinical Guidelines (CGs)**

**Category**	**Criteria**
1	CG based on the updating of previous NIHR HTA journal publication or publication(s)
2	CG drew on the evidence from previously published NIHR HTA funded studies. Criteria included:
	i) NIHR HTA study reviewed in detail
	ii) Table(s) taken from the NIHR HTA study and reproduced in CG
	iii) CG drew on NIHR HTA evidence for one or more sections of the CG
3	No NIHR HTA studies were cited

Assessment of the level of impact was performed independently by two researchers (ST and SB). Where there was disagreement, consensus was reached in consultation with a third researcher (AC). The agreement between the two researchers was assessed using a Cohen’s Kappa score.

## Results

### Phase 1

We identified a total of 122 NICE CGs issued between April 2001 and April 2012. Of these, 93 (76%) had referenced NIHR HTA funded studies (Table 2). We found inconsistency in the referencing style of the NIHR HTA journal publications making it difficult to accurately record all NIHR HTA journal publications cited. In order to identify peer reviewed articles cited in CGs we used an internal Evaluation, Trials and Studies Coordinating Centre (NETSCC) database which relies on the researchers informing the NIHR HTA programme when their articles are accepted for publication. We know that this does not always happen, so it is very likely that is an underestimation of the number of studies cited.

**Table 2:** **Impact of NIHR HTA funded studies on NICE CGs**

**Category**	**Number of CGs**	**Percentage**
1	3	2%
2	90	74%
3	29	24%
Total	122	

**Figure 1:**
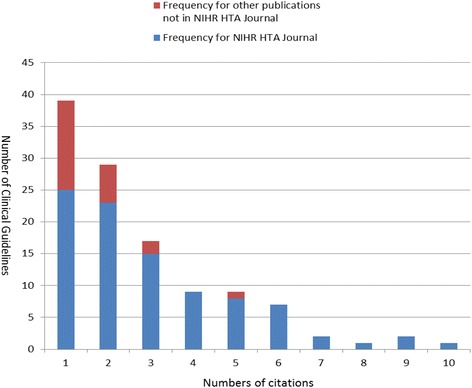
Number of publications cited in NICE Clinical Guidelines.

The total number of citations identified was 321. Of these, 284 were publications from the NIHR HTA journal series and 37 were publications in other peer reviewed journals, of which 5 were not in the NETSCC database and were identified by searching authors’ names and keywords. We were unable to identify any citations in 29 out of the 122 CGs. Figure 1 shows the numbers of citations of publications in the NIHR HTA journal and other publications in the CGs within the cohort.

### Phase 2

We assessed the impact on NICE CGs of NIHR HTA evidence and found that, of the 122 CGs, 3 (2%; CGs 7, 19, and 29) were included in category 1; 90 (74%) in category 2; and 29 (24%) in category 3 (Table 3). Agreement between the two researchers assessing impact was high (Kappa score = 0.980; weighted Kappa score = 0.980). Between the two researchers there was only disagreement over one CG, which was referred to the third researcher, and was then placed in category 2.

**Table 3:** **Impact of NIHR HTA studies on NICE Clinical Guidelines (CGs)**

**Category**	**Criteria**	**CGs**	Total (%)
1	CG based on the updating of previous NIHR HTA journal(s)	CG7, CG19, CG29	3 (2%)
2	CG drew on the evidence from previously published NIHR HTA funded studies	CG3, 8,9 10,11, 15,16, 17, 21, 25, 26, 27, 28, 30, 31, 32, 35, 36, 37, 38, 39, 42, 43, 44, 45, 47, 48, 49, 51, 52, 53, 54, 57, 58, 59, 61, 62, 63, 65, 66, 67, 68, 71, 72, 73, 74, 76, 77, 78, 79, 80, 81, 82, 85, 86, 87, 88, 89, 90, 91, 92, 93, 94, 95, 96, 97, 101, 102, 103, 105, 106, 107, 108, 109, 113, 117, 121, 122, 123, 124, 126, 127, 131, 132, 133, 135, 136, 137, 138, 139	90 (74%)
3	No NIHR HTA studies were cited	CG41, 50, 55, 56, 60, 64, 69, 70, 75, 83, 84, 98, 99, 100, 104, 110, 111, 112, 114, 115, 116, 118, 119, 120, 125, 128, 129, 130, 134	29 (24%)

In category 1, the CG was largely based on a previously published NIHR HTA journal publication or publications. For example, text in CG7 states that “*In April 2001, an HTA review was published on pressure-relieving devices for the prevention and treatment of pressure ulcers*” [19]. This review updated the earlier Cochrane systematic review, “*Beds, mattresses and cushions for pressure sore prevention and treatment*”. For the purposes of this guideline, the HTA review by Cullum et al. [19] was then updated by the Cochrane Wounds Group and National Collaborating Centre for Nursing and Supportive Care staff to provide the most up-to-date and rigorous source of clinical effectiveness evidence. For CG19, the methods used included “*Systematic review of the literature – to ‘update’ the previous Health Technology Assessment review on the clinical effectiveness and cost-effectiveness of routine dental checks*” [20].

Within category 2, the impact of NIHR HTA evidence on NICE CGs varied in the type and quantity of data used. The guideline could include a large volume of text about a particular study or studies; one or more tables of data; or contribute to meta-analysis. The evidence included may influence one particular section of the CG or relate to the whole CG. For example, in CG 95 [21] (*Chest Pain of Recent Onset*) the chapter on people presenting with acute chest pain was heavily influenced by NIHR HTA studies, tables of data from the original NIHR HTA sources were presented in the CG. In other cases, such as CG 137 [22] (*The epilepsies*), evidence from the SANAD trial [23] was used extensively throughout the CG, similarly in CG 108 [24] (*Chronic Heart Failure*) a systematic review funded by the NIHR HTA programme [25] provided data which had substantial impact on the CG as a whole. Initially we had planned to achieve a greater level of granularity, breaking down the data on impact into more categories; however, we found the ways in which the data was used by NICE was so heterogeneous (use of text, tables, trial data, meta-analyses in varying amounts and combinations) that this couldn’t be done. We therefore decided to include only 3 categories as described in Table 1, amalgamating the heterogeneous data into category 2.

## Discussion

This study assessed the percentage of NICE CGs informed by NIHR HTA studies and the impact of evidence from those studies on the included NICE CGs. The results show that NIHR HTA funded studies were referenced in 76% of NICE CGs and had an impact on 76% of the 122 NICE CGs. The study suggests that NIHR HTA research has a considerable contribution towards NICE CGs, and hence is well connected to both clinical practice and policy. Demonstrating or quantifying impact of health research is a complex issue [26] and not all the factors involved are readily quantifiable [16]; however, citation of health research studies in clinical guidelines has previously been used as an indicator of impact [15]. In this study, we found great heterogeneity in the way NIHR HTA evidence is used by NICE in CGs. Trying to quantify the impact of the different scenarios is both challenging and subjective.

It might be asked why NIHR HTA studies are not referenced in all CGs. It would be surprising, however, if there were a complete overlap in these portfolios as the remits of the NIHR HTA Programme and NICE are different. Both organisations were set up to support the NHS and patients in clinical decision-making – either by identifying important tractable research questions and hence filling evidence gaps (the NIHR HTA Programme) or by identifying areas of clinical uncertainty or inconsistency, and producing guidelines based on (among other things) available evidence to aid clinical decision-making (NICE). NICE commonly produces guidelines in areas where clinical practice is variable but the evidence base is clear. Once the evidence base has been established, the NIHR HTA Programme, by comparison, would not have so much to contribute in an area such as this. The NIHR HTA Programme will commission research in clinical areas which NICE may consider too small to devote the resources required to produce a guideline, but where there is clinical uncertainty resulting in possible sub-optimal patient care. A similar study conducted by Alderson and Tan [18] found that some Cochrane Review Groups did not have any Cochrane reviews cited by NICE CGs. It should also be remembered that the NICE guideline programme is not the NIHR HTA Programme’s only customer. NIHR HTA commissions work informed by research suggestions, and direct requests from other areas of NICE (such as the Interventional Procedures Advisory Committee) and other national bodies (such as the National Screening Committee).

The strengths of this study include its consideration of data over a long period of time, of more than 10 years, and its comparison of data from a major UK research funder with data from an organisation with an international reputation responsible for issuing national guidelines. Thus, this study gives an insight into the impact on clinical practice and policy, of the research which has been funded by the NIHR HTA Programme.

The limitations of this study include the time-limited nature of CGs, given that this is a snapshot view and some of the cohort considered herein have already been superseded, as well as the problem of accurately identifying all NIHR HTA sources used by NICE. NIHR HTA journal publications were not always cited in a consistent referencing style so we may have failed to identify some of these in the searches of the reference lists of CGs. Further, also we know that the data from our internal database used to identify other peer reviewed publications of NIHR HTA studies is incomplete. Additionally, this study does not include NIHR HTA studies which have been published after the relevant CG was issued; therefore, the contribution of NIHR HTA funded research to relevant clinical areas will have been underestimated.

This discussion of the interaction between the NIHR HTA Programme and NICE’s guidelines has, however, neglected the final common pathway. The NIHR HTA Programme strives to provide the NHS with the best possible evidence. NICE strives to provide it with the best possible guidance. We can see from this study that the interactions of these processes seem to work. What is missing is information on how new evidence or guidance [1,2,15,27,28] impacts on patient care and what factors influence the implementation of CGs [29‐32]. These relationships are not nearly so well understood and could be rich areas for future research. Additionally, the information from NICE has enabled us to assess how many publications were consulted in the preparation of the CGs; what we do not know is how this information was used in the guideline development process and what it was about the research that was most valued. It would be most informative to conduct qualitative research investigating the process of evidence use in guideline development.

## Conclusions

It is vitally important that there is a connection between health research and clinical practice and it is important to track the output of health research, especially its use in clinical guidelines. More than 75% of NICE CGs issued between April 2001 and April 2012 utilised evidence from NIHR HTA funded research. Findings suggest that evidence from NIHR HTA funded research impacts on NICE CGs and hence is well connected to both clinical practice and policy.

## Abbreviations

CG: Clinical guideline
HTA: Health technology assessment
NETSCC: Evaluation, Trials and Studies Coordinating Centre
NHS: National Health Service
NICE: National Institute for Health and Care Excellence
NIHR: National Institute for Health Research
